# Removal of Cu, Zn, Pb, and Cr from Yangtze Estuary Using the* Phragmites australis* Artificial Floating Wetlands

**DOI:** 10.1155/2017/6201048

**Published:** 2017-06-22

**Authors:** Xiaofeng Huang, Feng Zhao, Gao Yu, Chao Song, Zhi Geng, Ping Zhuang

**Affiliations:** ^1^Wuxi Fisheries College, Nanjing Agricultural University, Wuxi 214081, China; ^2^East China Sea Fisheries Research Institute, Chinese Academy of Fishery Sciences, Shanghai 200090, China; ^3^College of Life Science, East China Normal University, Shanghai 200241, China

## Abstract

Contamination of heavy metals would threaten the water and soil resources; phytoremediation can be potentially used to remediate metal contaminated sites. We constructed the* Phragmites australis* artificial floating wetlands outside the Qingcaosha Reservoir in the Yangtze Estuary. Water characteristic variables were measured in situ by using YSI Professional Pro Meter. Four heavy metals (copper, zinc, lead, and chromium) in both water and plant tissues were determined. Four heavy metals in estuary water were as follows: 0.03 mg/Kg, 0.016 mg/Kg, 0.0015 mg/Kg, and 0.004 mg/Kg. These heavy metals were largely retained in the belowground tissues of* P. australis*. The bioaccumulation (BAF) and translation factor (TF) value of four heavy metals were affected by the salinity, temperature, and dissolved oxygen. The highest BAF of each metal calculated was as follows: Cr (0.091 in winter) > Cu (0.054 in autumn) > Pb (0.016 in summer) > Zn (0.011 in summer). Highest root-rhizome TF values were recorded for four metals: 6.450 for Cu in autumn, 2.895 for Zn in summer, 7.031 for Pb in autumn, and 2.012 for Cr in autumn. This indicates that the* P. australis *AFW has potential to be used to protect the water of Qingcaosha Reservoir from heavy metal contamination.

## 1. Introduction

Accelerating economy and industrialization accompanied with vast consumption of toxic substances are an environmental contamination hazard. Heavy metal is considered as a kind of major toxic pollutants due to their persistence in the environment. It enters the aquatic ecosystems through natural geochemical process responding to human activities such as electroplating, smelting, sludge dumping, mining, intensive agriculture, and melting operations [1, 2]. In order to protect the creatures from heavy metal contamination, we should reduce the heavy metals from the contaminated areas.

There are three scientific methods to exact the heavy metal contamination: chemical methods, physical methods, and phytoremediation. Phytoremediation is a promising green technology because of its efficient capacity for removing various organic and inorganic pollutants. There are four strategies for plants to accumulate the heavy metals: phytoextraction, phytovolatilization, phytostabilization, and rhizofiltration. Phytoextraction is defined as the absorption and accumulation of heavy metals from the soil and water into the aboveground tissue of the plant [3, 4]. The uptake of pollutants from soil into the foliage followed by volatilization of the contaminants is called phytovolatilization [5], in which contaminated sites and sediments can be stabilized using vegetation, thereby mitigating the migration of toxic contaminants through the soil profile and reducing the risk of further environmental degradation [6]. Phytostabilization is defined as the fact that the heavy metals can be immobilized through the production of metallothioneins and phytochelatins [7]. The content of phytofiltration is defined as the roots of metal accumulating plants that absorb metals from polluted effluents and are later harvested to diminish the metals [8]. Of these four types of phytoremediation, phytoextraction is the most recognized approach which can be used for heavy metal removal from contaminated area. The remediation of heavy metal contaminated sites using plants was widely used in the heavy metal contaminated areas including urban storm water [9], agricultural fields [10], industrial units [11], mine tailings [12], and wastewater [13]. However, it is a valuable question whether this technology could be applied to extract the heavy metals in the estuary.

The artificial floating wetland (AFW) technology belongs to phytoremediation. The AFW is a promising green technology to extract the heavy metals from the contaminated areas, and it has also potential for providing significant wildlife habitat due to its high pollutant removal efficiency, easy operation and maintenance, and low energy requirements [14, 15]. In particular, some heavy metal contaminated areas were restored through adopting this green technology [16–18]. The heavy metals (e.g., Cd, Hg, Fe, Mn, Zn, and Cu) transferred from the substrata to the plant different tissues, and then they would be stored in plant tissues [19–21].

Although the AFW can be used to remediate metal contaminated area broadly, the design and operation of the AFW would be influenced by environment variables. First of all, the different type of macrophyte species can affect the efficiency of heavy metals removal [22]. The different type of aquatic macrophytes affected the redox status of the sediments by releasing oxygen from their roots into the rhizosphere, and the oxygen can help the wetland plant accumulate the heavy metals from all kinds of substrata [23]. The previous studies showed that the* P. australis* differed widely in their ability to accumulate heavy metals which make them able to be used in phytoremediation [24–26]. Secondly, the heavy metal accumulation was affected by other factors such as hydrologic regime, pollutant loading, temperature, and salinity [22, 27, 28]. Although the efficiency of heavy metal removal was affected by many environmental factors, the AFW was successfully constructed in lake and river to eliminate the polluted nutrition and heavy metals [15]. Therefore, both external (water-associated) and internal (root-associated) factors should influence the removal of heavy metal using AFW.

In order to protect the reservoir from the heavy metals contamination, the* P. australis* AFW was constructed outside the reservoir in the Yangtze Estuary. The four heavy metals (Cu, Zn, Pb, and Cr) in different tissues were measured in four seasons. The primary goals of the research were as follows: (1) to determine the capacity of four heavy metals in different tissues; (2) to compare the bioaccumulation factor and translocation factor of four metals; (3) to determine the relationship between water characteristic and heavy metals bioaccumulation in the* P. australis*.

## 2. Materials and Methods

### 2.1. Study Site

**Figure 1:**
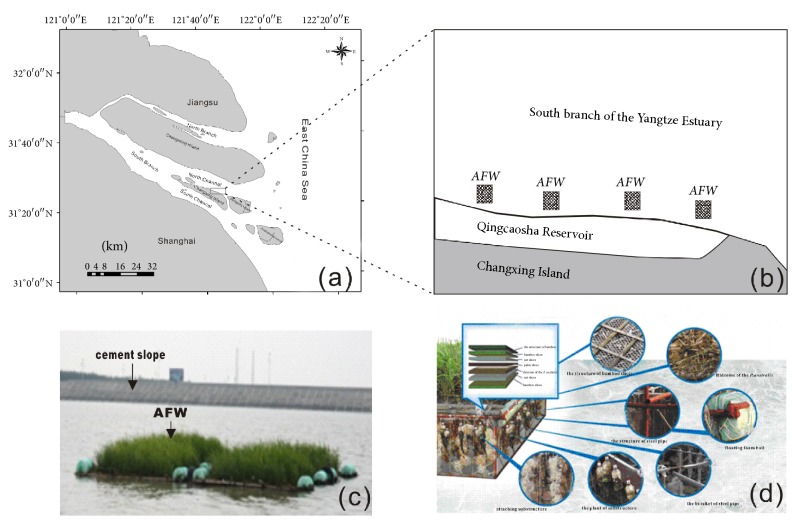
Location of the study area and the model of the* P. australis* AFW (AFW, artificial floating wetland; (a) the study site; (b) the* P. australis *AFW site; (c) the* P. australis* AFW photo; (d) the* P. australis* AFW model).

The experiment site was located outside the Qingcaosha Reservoir in the Yangtze Estuary (Figure 1(a)). The reservoir began to be built in 2004 and complicated in 2010, and it supplies drinking water for almost 11 million people in Shanghai. The cement dam prevents the water entering the reservoir directly from the Yangtze river, and it also prevents the salt marsh propagating along the dam.

### 2.2. Plant Material and AFW Construction

The* Phragmites australis* is a common macrophyte species along the Yangtze Estuary [29].

The* P. australis* AFW was constructed by both frame structure and* P. australis*. Each frame structure was divided into two parts: floating bed and artificial structure (Figures 1(c) and 1(d)). Each frame structure was 16 m^2^. Every experiment site was constructed by 12 fame structures; the area of each site included 200 m^2^ (Figure 1(b)). The* P. australis* AFW was fixed in the experiment sites in February 2014.

### 2.3. Analytical Procedures

Water characteristics including dissolved oxygen (DO, mg/L), salinity (SAL, *S*‰ mg/L), total dissolved solids (TDS, mg/L), specific conductance (SPC, us/cm), oxidation-reduction potential (ORP, mv), pH value, and temperature (*T*, °C) were measured in situ by using YSI Professional Pro Meter (YSI Inc., Ohio, USA). Water and* P. australis *(root, rhizome, and shoot) samples were collected at the stage of low tide in four seasons.

Water was sampled seasonally near the AFW surface (15 cm below the AFW) and put into plastic bottles and transported at 4°C. The water samples were immediately filtered through GF/C filters, acidified with HNO_3_ for preservation, and deposited in 50 mL tubes at −20°C in the laboratory. The determinations of Cu, Zn, Pb, and Cr in water were carried out by WGY-SIM cold atom fluorescence instrument (China national nuclear corporation). Each sample was analyzed in three replicates, and the results were given as mg/kg.

The* P. australis* specimens were divided into shoots, roots, and rhizomes separately in the laboratory. At first, in order to obtain the dry mass, the tissues were dried at 60°C for 72 h; A subsample (<1 g) of each dried sample was placed into a test tube for acid digestion. In the process of the acid digestion, ten milliliters of 55% nitric acid was added to samples and a 10 mL blank was then increased to 120°C and maintained for 3 h. After acid digestion, the plant samples were left to cool, and then diluted with distilled water to obtain a 20 mL sample. Finally, samples were filtered using 0.6 mm Whatman filter paper and 0.45 *μ*m cellulose nitrate membrane filter paper, a needle, and a syringe, after which they were stored in a refrigerator. Four heavy metals (Cu, Zn, Pb, and Cr) concentrations were determined using an inductively coupled plasma-atomic emission spectrophotometer (ICP-AES). The detection limit of four selected metals on the ICP-AES was 0.00001 ppm. Four metals concentrations were expressed as mg/kg dry mass.

### 2.4. Heavy Metals Bioaccumulation and Translation Factor

In order to differentiate the ability of the metals of subsequent translocation to the* P. australis* tissues and the value of the heavy metal accumulation, metal bioaccumulation factor (BAF) was calculated. Metal concentration ratio was expressed as water-to-root; water-to-rhizome; water-to-belowground parts, roots + rhizome; and water-to-shoot in the* P. australis.*

When the plant was used to accumulate heavy metals, translation factor (TF) of metals within the plant was evaluated [30]. The TF value could be expressed by the following ratio trace element: metal translocation factor (root-to-rhizome, root-to-shoot, and belowground parts-to-aboveground parts), and they were determined (TF = metal_[root]_/metal_[rhizome]_, or TF = metal_[root]_/metal_[shoot]_, or TF = metal_[below ground]_/metal_[above ground]_).

### 2.5. Data Analysis

The experiment data was expressed in the form of mean ± standard deviation. Statistical analysis was adopted by SPSS 20.0 software package. In order to analyze the relationship between the heavy metals uptake by the tissues of the plant and the environment factors, Pearson correlation coefficient can be calculated in this condition [31, 32]. Data was valued using Student's test for determining the significant change. The significance level was set at *P* < 0.05.

## 3. Results 

### 3.1. Environment Variables Varied in Four Seasons

Four heavy metals concentrations in water were generally ranked in the decreasing order: Zn > Cr > Cu > Pb (Table 1). The highest average temperature around the AFW presented in summer (26.82°C) and the temperature value showed low value in spring and winter (12.49°C and 15.16°C). The water pH was slightly acid in summer, except in spring, autumn, and winter when pH was alkalinity. The highest salinity was registered in autumn, while the lowest was in summer. The water in summer and autumn presented the low DO concentration value. However, the water was oxygenated in four seasons. The highest SPC presented in autumn was 917.44 ± 101.63 us/cm. The average of concentration of total phosphorus collected from the AFW surrounding area was between 0.04 mg/L and 0.11 mg/L, and total nitrogen was between 1.52 mg/L and 2.76 mg/L. The result showed that the environment variables around the AFW varied from one season to another season.

### 3.2. Heavy Metal Accumulated in the* P. australis* Tissues

**Figure 2:**
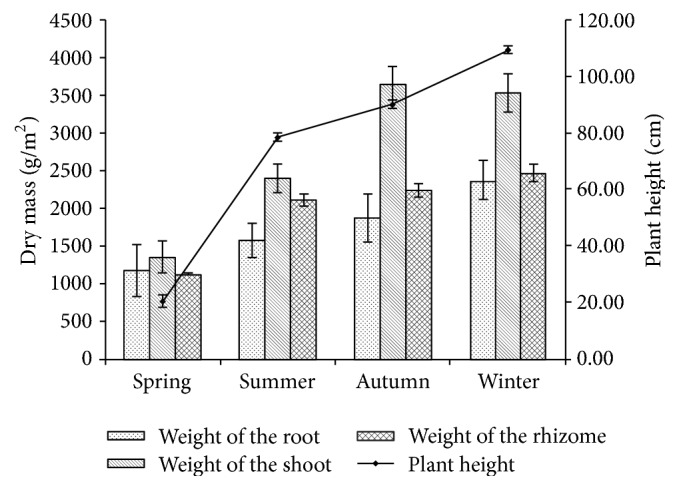
Dry weight of the* P. australis* aboveground tissues in four seasons.

After the* P. australis* tissues were collected from the AFW, then they were analyzed in the laboratory. The height of the* P. australis* kept increasing from the spring to winter, but the maxed height of the plant was no more than 110 cm (Figure 2). The different tissues dry mass were detected: the weight of shoot dry mass was higher than other tissues in the same season, and the highest weight of root (2371.2 g/m^2^), shoot (3640.3 g/m^2^), and rhizome (2463.4 g/m^2^) dry mass occurred in winter, autumn, and winter separately.

**Figure 3:**
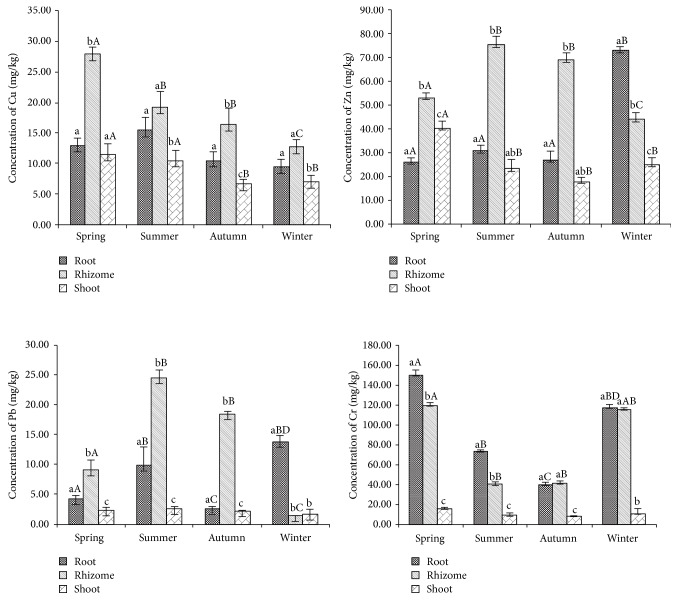
Metal concentration of Cu, Pb, Zn, and Cr in the* P. australis* shoots, roots, and rhizomes in four seasons (values are the means of six replicates ± standard deviation; significant difference between sites (within the same season) is showed by small letter; significant difference between seasons (within the same tissue) is showed by capital letter).

This experiment indicated* P. australis* capacities of heavy metals, and all different tissues (root, rhizome, and shoot separately) could concentrate the four kinds of heavy metals (Figure 3). Each heavy metal in the same tissue differed significantly from one season to another. Intertissues comparison at the same season revealed significant difference of the same heavy metal (Cu, Zn, Pb, and Cr separately). However, the interseasonal comparisons of the same tissue showed significant difference (Figure 3). The plant accumulated Cu in rhizome was significantly higher than that in its roots or shoots every season; the overall mean concentrations of Cu decreased in the following order: spring > summer > autumn > winter. The heavy metal Pb was detected in the different tissues: the rhizome was registered to have the highest Pb accumulation in summer (24.54 mg·kg^−1^ dry mass); on the other hand, the root accumulated the highest Pb (13.72 mg·kg^−1^ dry mass) in winter. Zn and Cr were two dominant elements whose value is reaching 43.9 and 41.58 mg·kg^−1^ dry mass in rhizome, 27.6 and 39.5 mg·kg^−1^ dry mass in root, and 17.5 and 8.14 mg·kg^−1^ dry mass in shoot, respectively. The rhizome accumulated Zn was higher than that in other tissues in spring, summer, and autumn. On the contrary, the root accumulated Zn was higher in winter than other rhizomes and shoots in winter. The root and rhizome exhibited the highest Cr (149.57 mg·kg^−1^ dry mass) accumulation followed by the above ground tissue. The* P. australis *accumulated significantly higher Cr in its root (149.58 mg·kg^−1^ dry mass) and rhizome (119.52 mg·kg^−1^ dry mass).

### 3.3. Heavy Metal Bioaccumulation and Their Translation Factor

The heavy metal largely accumulated in the belowground tissues, and therefore the BAF and TF values from one part to another part differed significantly of each heavy metal (Table 2). Interseasonal comparisons of Cu bioaccumulation factor at each tissue (water-to-root; water-to-shoot; belowground parts-to-aboveground parts) revealed no significant difference at the same season. However, the BAF value of Cu from water to rhizome significantly decreased with this order: autumn > summer > spring > winter. Interseasonal comparisons revealed no significant difference in Zn bioaccumulation factor within the same tissue (water-to-rhizome; water-to-shoot). However, the BAF value of Pb in the belowground tissue was significantly lower during spring and winter than that in summer and autumn. Interseasonal comparison within the same tissue revealed no significant difference in Pb bioaccumulation factor (water-to-root; water-to-below ground; water-to-shoot). However, the BAF value of Pb in the root was significantly higher in both spring and winter than that in both summer and autumn. Interseasonal comparison revealed significant difference in Cr bioaccumulation factor within the same tissue (water-to-root; water-to-rhizome; water-to-below ground). Summing up the results of Cr bioaccumulation factors, root exhibited the highest Cr bioaccumulation factor (0.046) followed by rhizome (0.037) and shoot (0.004) in spring.

Interseasonal comparisons within the same tissue revealed no significant difference in Cu and Zn transfer factor from belowground tissues to the aboveground tissue, and Cu and Zn translocation factor were observed from root to rhizome to be significantly lower than those in winter; however, the Cu translocation factor from root to shoot was significantly high in spring, and the Zn translocation factor from root to shoot was significantly different in four seasons. The highest translocation factor of Cu (below ground-above ground) was perceptible at the AFW site during winter (0.1694) followed by spring (0.1138), summer (0.1108), and then autumn (0.0858). Interseasonal comparisons revealed no significant difference in Cr transfer factor from root to shoot, the root-shoot TF value of Pb showed difference in four seasons.

## 4. Discussion and Conclusion

### 4.1. Metal Accumulation in the* P. australis* Tissues

The macrophyte tissues have the ability of accumulating and storing nutrients while they propagate and grow in aquatic ecosystem. Abundant heavy metals are essential elements to plant in the process of metabolism, so they can be detected in different tissues [33]. In the present study, the highest Pb concentration value in root (24.54 mg·kg^−1^) was observed, and it indicated that the studied species presented higher Pb concentrations in the roots than the range proposed by Noller et al. (1994) for uncontaminated freshwater plants (6.3–9.9 mg·kg^−1^) [34]. Among many other emergent vegetation types, the range of toxic level of Zn is below 230 mg·kg^−1^ in different tissues [30]. In this study, the Zn concentration in plants aboveground tissues was 66 mg·kg^−1^, and the highest Zn concentration (75.15 mg·kg^−1^) was found in rhizome in summer, so the element Zn value in shoot was lower than the concentration already mentioned. Among the studied heavy metals, the element Cu is also an essential heavy metal to plant, but it has toxic effects when shoots and leaves accumulated Cu in concentrations exceeding 20 mg·kg^−1^ [31]. However, the highest Cu concentration (27.84 mg·kg^−1^) was found in the rhizome in summer, and the lowest Cu concentration (6.96 mg·kg^−1^) was found in shoot in winter. Smiri et al. (2015) [13] studied the* P. australis* which accumulated the highest amount of Cr in the roots (1,800 mg Cr/kg dry tissue), compared with 149.58 mg Cr/kg in the roots. The results indicated that the* P. australis *that grew in the AFW usually contained lower concentration than this threshold. Four heavy metals accumulated in the different tissues of the* P. australis*, and the bioaccumulation value of the heavy metal was lower than the result of the other plant.

It is an efficient strategy for plants to be considered a “root accumulator” of metals. Numerous studies found that various wetland plants actually accumulate and immobilize certain metals in their root tissues, thus limiting distribution to aboveground parts [35, 36]. Bioaccumulation of the heavy metals in the roots is a strategy that the plant can restrict distribution of heavy metals to the aboveground tissues [37, 38]. In the current study, the belowground tissues accumulate higher concentrations of the four metals than that in the aboveground tissue (shoot), which indicated the* P. australis* accumulated heavy metals in the brackish water. The tissues differed widely in their ability to accumulate heavy metals in every season (e.g., in spring, root concentrate 12.91 mg·kg^−1^ Cu, 26.13 mg·kg^−1^ Zn, 4.22 mg·kg^−1^ Pb, and 149.58 mg·kg^−1^ Cr). This conclusion easily explained that Cu, Zn, Pb, and Cr are essential micronutrients for the different tissues.

### 4.2. Removal of Heavy Metals Was Affected by the Water Characteristic

Heavy metal bioaccumulation was largely influenced by both external (water-associated) and internal (root-associated) factors. The ability of heavy metals accumulation and translocations is influenced by the following factors: the variations in plant species, the growth stage of the plants, and water characteristics control absorption. In particular, the influence of season variation on the heavy metal removal by the plant has consistently been reported [39, 40]. The various environment factors (such as pH, temperature, dissolved oxygen, redox potential chemical speciation, sediment type, and salinity) can obviously influence the heavy metal bioavailability [37, 38]. Hydraulic conditions can strongly influence the ability of heavy metal removal through the AFW technologies [41]. In the present study, Pearson correlation coefficient between metals concentrations in the tissues and water factors was calculated for determination of relationship between plants and water factors (e.g., DO, pH, temperature, and salinity) (Table 4).

When the* P. australis* grew in higher pH conditions (<6.0), the root reduced the uptake of many metals, but the high pH did not prevent the absorbing process of Cu (Batty et al. [42]). Although the water pH value below the AFW exceeded this value (6.0) (Table 4), it was not significantly different than the value of TF for selected metals, and the tissues showed the highest TF of the selected metals in spring (Table 3). The dissolved oxygen is another important environment variable which can affect the removal of heavy metals [43]. The dissolved oxygen has a positive effect on Cr concentration about the* P. australis* underground tissues, respectively, and negative relationships have been observed in the aboveground tissue. The ability of absorbing other heavy metals (Cu, Zn, and Pb) was not affected by the water dissolved oxygen in four seasons. Salinity was another major environmental factor limiting plant growth and productivity [44, 45]. The aboveground tissues of the* P. australis* had negative correlation between salinity values and Cu/Zn contents, and salinity also had a positive effect on Cr uptake in the aboveground tissue.

### 4.3. Applying AFW to Remove the Heavy Metal in Estuary

The majority of estuary district has become an economic and large population area all over the world in recent decades [46]. As a result, the estuary ecosystem is strongly influenced by human settlements, agriculture, and industry. For example, the Yangtze river has become a main heavy metals receiving and capturing area when the heavy metal pollutants coming from the industrial waste and residential waste pooled into this river [47, 48]. Because the heavy metal contaminated the water and soil, the organic tissues of the fish would accumulate the heavy metals through the biological concentrations [49].

In order to eliminate heavy metals from the environment, how to remove the heavy metal from estuary water becomes an important topic of study. The salt marshes (*Phragmites australis*,* Spartina alterniflora*, and* Scirpus mariqueter*) could accumulate the heavy metals (e.g., Cu, Zn, and Pb) from the substrate or water in the Yangtze Estuary [50]. However, the areas of salt marsh have significantly been reduced due to the estuary environmental problems (e.g., human activities, local industrialization, and urbanization) in the past decades [51–54]. In particular, the cement was used to construct the dam along the reservoir, and the* P. australis *and other salt marsh plants cannot survive in this area. However, the* P. australis* community played some typical ecological functions, such as giving habitat and ecological service. This marsh plant can remove the heavy metals and radionuclides in the environment, especially in a large scale area and low concentration of pollution sites. According to the different capacities of metal uptake, the* P. australis *was able to accumulate relatively heavy metals concentrations in the aboveground tissues which could be good candidates for phytoremediation.

In this experiment, the small scales AFW was successfully constructed in the estuary, and the heavy metals were also concentrated in the different tissues. It indicated that this technology presents sustainable use of natural and/or constructed ecosystems for environmental protection and restoration. The current study implied that the* P. australis *AFW has enough potential to be used for heavy metals contaminated area along the estuary.

## Figures and Tables

**Table 1:** Waters characteristics around the AFW in different seasons (mean ± SD).

Characteristics	Spring (*n* = 3)	Summer (*n* = 3)	Autumn (*n* = 3)	Winter (*n* = 3)
	Mean ± SD	Mean ± SD	Mean ± SD	Mean ± SD
*T* (°C)	12.49 ± 1.92	26.82 ± 1.11	23.69 ± 0.26	15.16 ± 0.44
DO (mg/L)	10.36 ± 1.39	6.59 ± 0.49	6.36 ± 0.49	9.69 ± 0.31
Salinity (*S*‰)	1.18 ± 0.01	2.15 ± 0.06	3.53 ± 0.12	1.24 ± 0.01
TDS (mg/L)	248.95 ± 14.21	217.89 ± 45.73	717.42 ± 184.1	321.26 ± 18.39
SPC (us/cm)	382.9 ± 21.64	332.34 ± 70.31	917.44 ± 101.63	489.66 ± 27.52
ORP (mv)	115.78 ± 13.62	92.99 ± 75.18	128.06 ± 23.11	85.17 ± 48.59
pH	7.62 ± 1.19	6.67 ± 0.92	8.71 ± 0.47	7.84 ± 0.03
Total nitrogen	2.41 ± 0.85	2.57 ± 0.43	2.76 ± 0.75	2.63 ± 0.48
Total phosphorus	0.12 ± 0.07	0.11 ± 0.04	0.09 ± 0.03	0.04 ± 0.02
Metals (mg/Kg)				
Cu	0.003 ± 0.001	0.003 ± 0.002	0.002 ± 0.001	0.003 ± 0.001
Zn	0.014 ± 0.008	0.017 ± 0.008	0.02 ± 0.008	0.014 ± 0.003
Pb	0.001 ± 0.001	0.002 ± 0.001	0.002 ± 0.001	0.002 ± 0.001
Cr	0.005 ± 0.001	0.008 ± 0.002	0.004 ± 0.001	0.003 ± 0.001

**Table 2:** Mean values and standard deviation of heavy metals (Cu, Zn, Pb, and Cr) bioaccumulation factor (BAF) (metals concentration ratio of water-root; water-rhizome; water-belowground parts, roots + rhizome; and water-shoot) in *P*. *australis*.

Metals		Spring	Summer	Autumn	Winter
		Mean ± SD	Mean ± SD	Mean ± SD	Mean ± SD
Cu	Water-root	0.001 ± 0.000	0.007 ± 0.005	0.007 ± 0.003	0.015 ± 0.011
	Water-rhizome	0.035 ± 0.169^a^	0.043 ± 0.038^a^	0.047 ± 0.020^a^	0.006 ± 0.004^b^
	Water-belowground parts	0.036 ± 0.175	0.050 ± 0.044	0.054 ± 0.023	0.022 ± 0.015
	Water-shoot	0.004 ± 0.001	0.005 ± 0.007	0.004 ± 0.002	0.003 ± 0.002
Zn	Water-root	0.006 ± 0.007^a^	0.001 ± 0.001^b^	0.001 ± 0.001^b^	0.005 ± 0.001^ab^
	Water-rhizome	0.005 ± 0.002	0.005 ± 0.004	0.004 ± 0.002	0.003 ± 0.000
	Water-belowground parts	0.011 ± 0.006^a^	0.007 ± 0.005^b^	0.005 ± 0.003^ab^	0.008 ± 0.0023^b^
	Water-shoot	0.003 ± 0.002	0.001 ± 0.001	0.001 ± 0.000	0.001 ± 0.000
Pb	Water-root	0.003 ± 0.001	0.006 ± 0.003	0.001 ± 0.000	0.010 ± 0.005
	Water-rhizome	0.007 ± 0.003^a^	0.016 ± 0.011^b^	0.012 ± 0.004^b^	0.001 ± 0.000^c^
	Water-belowground parts	0.013 ± 0.003	0.011 ± 0.001	0.010 ± 0.000	0.011 ± 0.001
	Water-shoot	0.001 ± 0.000	0.001 ± 0.000	0.001 ± 0.000	0.001 ± 0.000
Cr	Water-root	0.046 ± 0.014^a^	0.010 ± 0.004^b^	0.004 ± 0.001^b^	0.045 ± 0.007^ab^
	Water-rhizome	0.037 ± 0.009^a^	0.007 ± 0.007^b^	0.009 ± 0.002^ac^	0.045 ± 0.009^abc^
	Water-belowground parts	0.083 ± 0.023^a^	0.017 ± 0.010^b^	0.013 ± 0.004^b^	0.091 ± 0.014^ab^
	Water-shoot	0.004 ± 0.001	0.001 ± 0.001	0.001 ± 0.000	0.004 ± 0.002

Values are the means of six replicates ± standard deviation. Significant difference between seasons (within the same tissue) is showed by small letter.

**Table 3:** Mean values and standard deviation of metals translation factor (TF) (metals concentration ratio of root-rhizome and root-leaf and belowground parts and root + rhizome-aboveground parts and shoot) in *P. australis*.

Metals		Spring	Summer	Autumn	Winter
		Mean ± SD	Mean ± SD	Mean ± SD	Mean ± SD
Cu	Root-rhizome	3.715 ± 1.653^a^	5.257 ± 0.916^b^	6.450 ± 0.663^b^	0.433 ± 0.028^c^
	Root-shoot	4.611 ± 2.094^a^	0.710 ± 0.438^b^	0.642 ± 0.130^b^	0.243 ± 0.063^b^
	Below ground- above ground	0.113 ± 0.017	0.110 ± 0.060	0.085 ± 0.012	0.169 ± 0.042
Zn	Root-rhizome	0.829 ± 0.122^a^	2.895 ± 0.331^b^	2.589 ± 0.414^b^	0.602 ± 0.0302^c^
	Root-shoot	0.615 ± 0.122^a^	0.915 ± 0.176^b^	0.779 ± 0.247^c^	0.343 ± 0.037^d^
	Below ground- above ground	0.336 ± 0.006	0.238 ± 0.056	0.186 ± 0.060	0.214 ± 0.023
Pb	Root-rhizome	2.148 ± 0.220^a^	2.913 ± 1.695^a^	7.031 ± 0.529^b^	0.101 ± 0.0043^c^
	Root-shoot	0.581 ± 0.151^a^	0.280 ± 0.006^b^	0.822 ± 0.232^a^	0.120 ± 0.058^b^
	Below ground- above ground	0.186 ± 0.051^a^	0.077 ± 0.015^b^	0.104 ± 0.034^b^	0.109 ± 0.052^b^
Cr	Root-rhizome	0.810 ± 0.056^a^	0.599 ± 0.387^a^	2.102 ± 0.135^b^	0.987 ± 0.0123^a^
	Root-shoot	0.109 ± 0.038	0.130 ± 0.046	0.418 ± 0.049	0.093 ± 0.048
	Below ground- above ground	0.133 ± 0.039^a^	0.306 ± 0.212^a^	0.200 ± 0.036^a^	0.093 ± 0.0475^b^

Values are the means of six replicates ± standard deviation. Significant difference between seasons (within the same tissue) is showed by small letter.

**Table 4:** Pearson correlation coefficients between metal concentrations in the aboveground and underground tissues and water factors.

Tissues	Season	Metals	Water factors						
			DO	SAL	TDS	SPC	ORP	PH	*T*
Aboveground	Spring	Cu	NS	NS	NS	NS	NS	NS	NS
		Zn	NS	NS	0.701^*∗*^	0.699^*∗*^	NS	NS	NS
		Pb	NS	NS	NS	NS	NS	NS	NS
		Cr	NS	NS	NS	NS	NS	NS	NS
	Summer	Cu	NS	−0.750^*∗*^	−0.675^*∗*^	−0.672^*∗*^	NS	NS	NS
		Zn	NS	−0.767^*∗*^	−0.680^*∗*^	−0.679^*∗*^	NS	NS	NS
		Pb	NS	NS	NS	NS	NS	NS	NS
		Cr	NS	NS	NS	NS	NS	NS	NS
	Autumn	Cu	NS	−0.778^*∗*^	NS	NS	NS	NS	NS
		Zn	NS	−0.787^*∗*^	NS	NS	NS	NS	NS
		Pb	NS	NS	NS	NS	NS	NS	NS
		Cr	−0.831^*∗∗*^	NS	0.835^*∗∗*^	0.836^*∗∗*^	0.744^*∗*^	−0.720^*∗*^	NS
	Winter	Cu	NS	−0.751^*∗*^	NS	NS	NS	NS	NS
		Zn	NS	−0.442	NS	NS	NS	NS	NS
		Pb	NS	−0.684^*∗*^	NS	NS	NS	NS	NS
		Cr	NS	0.668^*∗*^	NS	NS	NS	NS	NS
Underground	Spring	Cu	NS	NS	NS	NS	NS	NS	NS
		Zn	NS	NS	NS	NS	NS	NS	NS
		Pb	NS	NS	NS	NS	NS	NS	NS
		Cr	NS	NS	NS	NS	NS	NS	NS
	Summer	Cu	NS	NS	NS	NS	NS	NS	NS
		Zn	NS	NS	NS	NS	NS	NS	NS
		Pb	NS	NS	NS	NS	NS	NS	NS
		Cr	NS	NS	NS	NS	NS	NS	NS
	Autumn	Cu	NS	NS	NS	NS	NS	NS	NS
		Zn	NS	NS	NS	NS	NS	NS	NS
		Pb	NS	NS	NS	NS	NS	NS	NS
		Cr	0.534^*∗*^	NS	NS	NS	NS	NS	NS
	Winter	Cu	NS	NS	NS	NS	NS	NS	NS
		Zn	NS	NS	NS	NS	NS	NS	NS
		Pb	NS	NS	NS	NS	NS	NS	NS
		Cr	NS	NS	NS	NS	NS	NS	NS

NS: no significant correlation. ^*∗*^Correlation is significant at the 0.05 level. ^*∗∗*^Correlation is significant at the 0.01 level.
